# Assessing Genome-Wide Diversity in European Hantaviruses through Sequence Capture from Natural Host Samples

**DOI:** 10.3390/v12070749

**Published:** 2020-07-11

**Authors:** Melanie Hiltbrunner, Gerald Heckel

**Affiliations:** 1 Institute of Ecology and Evolution, University of Bern, 3012 Bern, Switzerland; melanie.hiltbrunner@iee.unibe.ch; 2 Swiss Institute of Bioinformatics, Quartier Sorge, 1011 Lausanne, Switzerland

**Keywords:** hybrid sequence capture, virus genomes, targeted enrichment, high-throughput deep sequencing, *de novo* assembly, hantavirus phylogeny, evolutionary history, rodent-borne viruses

## Abstract

Research on the ecology and evolution of viruses is often hampered by the limitation of sequence information to short parts of the genomes or single genomes derived from cultures. In this study, we use hybrid sequence capture enrichment in combination with high-throughput sequencing to provide efficient access to full genomes of European hantaviruses from rodent samples obtained in the field. We applied this methodology to Tula (TULV) and Puumala (PUUV) orthohantaviruses for which analyses from natural host samples are typically restricted to partial sequences of their tri-segmented RNA genome. We assembled a total of ten novel hantavirus genomes *de novo* with very high coverage (on average >99%) and sequencing depth (average >247×). A comparison with partial Sanger sequences indicated an accuracy of >99.9% for the assemblies. An analysis of two common vole (*Microtus arvalis*) samples infected with two TULV strains each allowed for the *de novo* assembly of all four TULV genomes. Combining the novel sequences with all available TULV and PUUV genomes revealed very similar patterns of sequence diversity along the genomes, except for remarkably higher diversity in the non-coding region of the S-segment in PUUV. The genomic distribution of polymorphisms in the coding sequence was similar between the species, but differed between the segments with the highest sequence divergence of 0.274 for the M-segment, 0.265 for the S-segment, and 0.248 for the L-segment (overall 0.258). Phylogenetic analyses showed the clustering of genome sequences consistent with their geographic distribution within each species. Genome-wide data yielded extremely high node support values, despite the impact of strong mutational saturation that is expected for hantavirus sequences obtained over large spatial distances. We conclude that genome sequencing based on capture enrichment protocols provides an efficient means for ecological and evolutionary investigations of hantaviruses at an unprecedented completeness and depth.

## 1. Introduction

Knowledge about the diversity and evolution of viruses has strongly benefitted from the improvement of detection methods and access to genetic and genomic information. Sequence data may sometimes represent the only information about a novel virus (e.g., [1]) but even for relatively well-known taxa, this information is often restricted to short parts of the genomes or single complete genomes derived from cultures (e.g., [2,3]). This hampers progress in the development of diagnostic tools and in taxonomic classification that remains sometimes ambiguous without access to full genome information (e.g., [4]). Sequencing complete virus genomes with classical methods represents a significant investment of time and effort (see, e.g., [5,6]). As a consequence, the extent and distribution of genomic diversity within many virus taxa remains unknown to date. High-throughput sequencing methods have started to remedy this and enabled characterization of virus diversity at unprecedented scales (e.g., [7,8]). Access to entire RNA virus genomes, however, is still challenging particularly for many samples obtained in the field because of technical challenges posed by loss of RNA integrity and low viral loads (e.g., [7,9,10]). Increasing the proportion of virus RNA for sequencing using cell culture is often difficult and may result in genetic variation that is not representative of the natural sample. Therefore, using total RNA extracted directly from natural host tissue is preferable in order to obtain evolutionarily relevant information on the virus genomes [11].

Here we adapt hybrid sequence capture to obtain multiple novel genome sequences of two orthohantavirus species from natural host tissues sampled in the field. Hybrid sequence capture was developed to enrich rare target nucleic acid sequences in problematic samples and has been used, e.g., for the enrichment of ancient DNA [12,13], bacteria [14] and viruses [7,15,16]. The application of hybrid sequence capture promises a higher efficiency of high-throughput sequencing for virus genomes compared to shotgun approaches and accordingly a massive reduction in the costs per genome [17]. Furthermore, optimized designs for sequence capture baits allow now in principle the coverage of full genomes and the enrichment of complex metagenomic samples [18]. However, for each new bait design, it remains unclear *a priori* how strongly the efficiency of a design is impeded by genetically diverse capture targets and whether enough target sequence data can be captured for the reconstruction of full virus genomes. It has been shown that sequence divergence of 25% and more between bait and target worked for enrichment [18] and specific targets with up to 58% divergence have been captured [19,20].

Here, we explore the potential of hybrid sequence capture for hantavirus genome recovery in Tula (TULV) and Puumala virus (PUUV), two vole-borne orthohantaviruses circulating in many regions of Europe with very high genetic diversity. Orthohantaviruses (family *Hantaviridae*) are single-stranded, negative-sense RNA viruses with many recent discoveries resulting in a dynamic taxonomy [4]. The genome of about 12 kilobase pairs is subdivided into three segments, of which the small segment (S-segment) encodes the nucleocapsid protein and a non-structural protein with a function that is not fully characterized in a second overlapping reading frame [21]. The medium segment (M-segment) codes for the glycoproteins (Gn and Gc), which function as surface proteins and interact with the host cell receptors for initial infection. The large segment (L-segment) encodes the RNA-dependent RNA polymerase [21]. Due to the error-prone replication by the RNA polymerase, the mutation rates in hantaviruses are typically very high [4,22,23]. At the regional and continental geographic scale, this has been shown to lead to very strong mutational saturation in hantavirus sequence datasets resulting in too young age estimates and potentially biased phylogenetic analyses [24]. Full genome sequences are preferable to partial segments to most accurately determine evolutionary relationships and to assess the importance of recombination, reassortment and adaptive processes in the history of hantaviruses [23,24,25], but, to date, efficient protocols for genomic sequencing are not available.

The publicly available PUUV genomes and the TULV reference genome were sequenced with classical methods (see below), while only recently was high-throughput sequencing based on shotgun approaches used to generate additional TULV genomes [9,26]. In a study of adaptive evolution between two phylogenetic clades within TULV [26], 12 TULV genomes were assembled from shotgun sequencing data but with very low efficiency (proportion of virus reads between 0.001 and 0.2%). Here, we use hybrid sequence capture to resolve two double-infections with TULV from natural samples at the genome level, complement this with additional genomes from the same phylogenetic clades for further comparison and assess the applicability of our approach further by sequencing more genetically divergent PUUV genomes from different localities. We use the novel TULV and PUUV genomes together with publicly available complete sequences to investigate the genomic landscape, to compare the extent of divergence between genomic segments and assess the impact of genome scale data for the reconstruction of phylogenetic relationships.

## 2. Materials and Methods

### 2.1. Virus Samples, RNA Extraction and Sanger Sequencing

**Figure 1:**
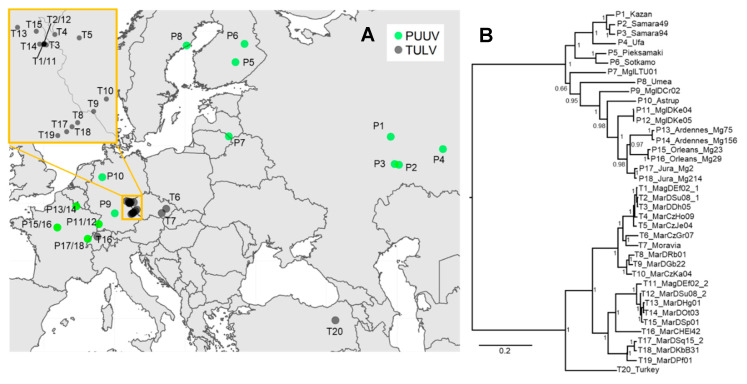
(**A**) Map of Europe showing the origin of the novel and previously published Tula (TULV) (T#) and Puumala (PUUV) (P#) genome sequences analyzed in this study (for details see Table S1). The insert shows a zoom into the contact region between two phylogenetic clades in TULV from where two double-infected individuals (T1/11 and T2/12) were sequenced (see [26]). (**B**) Phylogenetic tree based on the concatenated complete coding nucleotide sequence (CDS) of TULV and PUUV. Posterior probabilities of Bayesian analyses are given for major nodes. Sequence labels follow Table S1.

We used samples from TULV-positive common voles and PUUV-positive bank voles from different locations in Central Europe, chosen to cover the high genetic variability in the region, together with published genome data (Figure 1, Table 1, Table S1; see [24]). Total RNA was extracted from lung tissue used in previous studies with a modified QIAzol protocol as described in [27] and partial S-segment sequences were generated with Sanger sequencing [23,24,28]. RNA concentration was measured for each sample using the Qubit RNA BR Assay Kit (Invitrogen, Basel, Switzerland) and RNA quality was determined on a Fragment Analyzer CE12 (Advanced Analytics, Agilent, Santa Clara, CA, USA).

### 2.2. Library Preparation

Sequencing libraries were prepared using the NEBNext Ultra RNA Library Prep Kit for Illumina (New England Biolabs, Ipswich, USA) with the NEBNext Multiplex Oligos Dual Index Primers Set 1 (New England Biolabs) for indexing or using the TruSeq RNA-library Kit from Illumina. TruSeq libraries were prepared according to the standard protocol with fragmentation for 40 s at 94 °C. Most of the original samples already had an RNA integrity number (RIN) < 2, indicating high fragmentation. Therefore, the libraries were prepared according to the NEBNext protocol for highly degraded RNA. Nuclease-free low-bind tubes (MAXYmum Recovery, Axygen, Union City, CA, USA) were used at any time during NEBNext library preparation. Thermal incubation steps were performed on the same PCR machine (Veriti, Applied Biosystems, Waltham, Massachusetts, USA). Library clean-up was done as described in the NEBNext RNA library prep protocol using the recommended AMPure XP beads (Agencourt, Beckman Coulter, Nyon, Switzerland). The final amplification step of both sequencing library types (NEBNext and TruSeq) was performed with 15 PCR cycles following the manufacturer’s recommendation to ensure enough input material for hybrid sequence capture. Library quality was assessed on a Fragment Analyzer and concentration was measured with Qubit DNA BR Assay Kit (Invitrogen). The concentrations were between 44 and 165 ng/μL. For one sample (MagDEf02), a NEBNext and a TruSeq sequencing library was made and sequence capture was performed twice for each. For one sample (MarDSu08), two NEBNext libraries were prepared and captured 1× or 2× (see below).

### 2.3. Hybrid Sequence Capture

The sequencing libraries were enriched for TULV or PUUV using the In-Solution Sequence Capture for Targeted High-Throughput Sequencing Kit from MYbaits (MYcroarray, Arbor Biosciences, Ann Arbor, MI, USA). We provided 91 published sequences to MYcroarray for the specific capture bait design. We included mainly TULV sequences including partial Adler virus sequences but also a Prospect Hill virus and 4 PUUV genomes (Table S2). For our design, we chose short (80 nt) RNA baits to reduce the risk of secondary structure formation. There were 8489 individually designed baits in the final design (Table S5).

We used up to 2 μg of each library for sequence capture because in a previous experiment, the shotgun-sequencing of six TULV libraries each with Illumina MiSeq and HiSeq technology resulted in low virus sequence yield (MiSeq average: 0.014%, HiSeq average: 0.05%; [26]). To avoid index dissociation via jumping PCR during library amplification [29,30] each library was captured in an individual reaction with 1/6 of the default amount of sequence baits. For sequence capture, all libraries were incubated at 55 °C for 40 h. After clean-up, the enriched libraries were amplified using NEBNext Ultra II Q5 Master Mix (New England Biolabs) and library specific P5 and P7 primers [31]. AMPure XP beads were used for PCR clean-up. Four sequence libraries were additionally captured a second time at 65 °C for 40 h to potentially increase the virus sequence yield. The final library qualities and concentrations were assessed on a Fragment Analyzer and a Qubit.

### 2.4. Sequencing and Hantavirus Genome Assembly

The libraries were sequenced on a HiSeq3000 2 × 150 bp or on a MiSeq 2 × 300 bp (Illumina, San Diego, CA, USA) on the Next Generation Sequencing Platform of the University of Bern. Reads were trimmed with Trimmomatic-0.36 and paired and unpaired reads were sorted into separate files. For the assembly, only paired reads were used. Identical sequences were removed using PRINSEQ [32] with -derep set to 1. The Iterative Virus Assembler (IVA) [33] was used for *de novo* assembly with default parameters. The resulting contigs were compared against sequences on GenBank for identification using the BLAST algorithm. Virus contigs were aligned in BioEdit [34] and overlapping contigs were merged to a consensus sequence. Sequence reads from each library were then back-mapped against the *de novo* virus consensus genome in order to receive mapping statistics and to control sequence quality as suggested by [35]. For back-mapping, a Burrows–Wheeler Aligner (BWA) was used for read alignment [36], GATK v3.7.0 [37] to create vcf (variant call format) and consensus sequences and samtools [38] to generate bam files which contained only mapped reads and to calculate mapping statistics. In R [39], the average coverage of each genome and the minimal and maximal coverage of each segment were calculated using the output of the back-mapping (Table 1). GenBank accession numbers for sequence data are provided in Table S1. To estimate multiplexing levels for future experiments and to test the input limits of IVA to successfully assemble virus genomes *de novo*, the library with the fewest reads (MarCzGr07) was randomly divided into four parts using fastq-splitter v0.1.2 (http://kirill-kryukov.com/study/tools/fastq-splitter/) prior to deduplication with PRINSEQ. Each part was then assembled individually with IVA.

### 2.5. Phylogenetic Analyses

Phylogenetic reconstructions were based on the complete coding sequences (CDS) of TULV and PUUV without the highly variable flanking regions in S- and M- genome segments, for which proper alignment is challenging. Phylogenetic trees were inferred for nucleic acid sequences in MrBayes v.3.2.6 [40] on CIPRES [41] using mixed models. Individual nucleotide substitution rate priors in MrBayes were used (see [24]). Nucleotide sequence data were partitioned into 1st + 2nd and 3rd codon position with the evolutionary rate unlinked across partitions. Four independent analyses were performed each with 100,000 generations of Markov chain Monte Carlo chains and sampling every 100 generations. The segments were considered as independent genes in the model using partitioning in MrBayes. The trees were visualized in FigTree v. 1.4.2 [42].

### 2.6. Multidimensional Scaling, Recombination and Isolation-By-Distance Analyses

For TULV and PUUV, pairwise genetic distances (p-distances) were inferred in MEGA7 [43] including transitions and transversions and assuming uniform mutation rates among sites. Alignment gaps were excluded pairwise. For visualization, multidimensional scaling was performed on the genetic distance matrix of sequences of the three genome segments using the cmdscale command in R. Genomes were tested for recombination with a full exploratory recombination scan in RDP4 [44] using all available methods. Likely recombination events were reanalyzed with all methods and significance levels reported from MaxChi analogous to [26]. Isolation-by-distance analyses relating genetic distances or branch lengths from phylogenetic analyses and spatial distances between sequence pairs were performed as described in [24]. Branch lengths were extracted from the phylogeny using the cophenetic command in R. A Mantel test was performed between genetic and spatial distance matrices to assess the statistical significance of the association.

### 2.7. Nucleotide Diversity and Divergence between Phylogenetic Clades

For TULV and PUUV, the overall average nucleotide diversity for each genome segment was calculated in R as the average of the pairwise p-distance between sequences. Additionally, the nucleotide diversity along each genome segment and average number of nucleotide substitutions per site between hantavirus species (D_XY_) was calculated using a sliding-window approach implemented in DnaSP [45]. The window length was set to 100 nucleotides (nt) and the step size to 25 nt. The mean average divergence and the net average divergence between species were calculated in MEGA7.

## 3. Results

### 3.1. Hybrid Sequence Capture and Sequencing of TULV and PUUV Genomes

**Figure 2:**
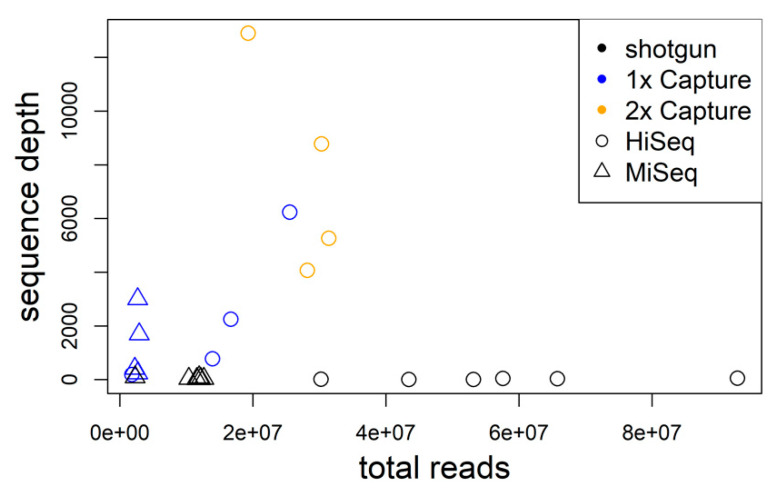
The average genome sequence depth significantly increases with the number of total sequence reads for libraries enriched using hybrid capture. Shotgun data are shown for comparison in black.

Hybrid sequence capture resulted in comparatively large numbers of hantavirus sequence reads for each wild rodent sample. On average, 1.6 × 10^5^ reads from 1× captured libraries and 6.4 × 10^5^ reads from 2× captured libraries back-mapped to TULV/PUUV compared to 9.3 × 10^3^ TULV reads obtained from shotgun sequencing ([26]; Table 1, Figure 2). The proportion of virus reads (deduplicated reads post-mapping) for 1× captured libraries was 0.45–5.7% and 1.19–5.53% for 2× captured libraries. In comparison to TULV shotgun sequencing, the average proportion of virus reads was 120-fold larger in 1× captured libraries and 184-fold in 2× captured libraries. For sample MarDSq15, 1× sequence capture yielded 515,194 TULV reads (2.015% of all reads) while shotgun sequencing resulted in 583 reads (0.001%), equivalent to a 2000-fold enrichment (Table 1). On average, two capture rounds yielded 1.5 times higher enrichment than one capture round. The two libraries of MarDSu08 that were captured 1× and 2× had similar proportions of TULV reads (1.07× enrichment).

The average virus genome sequencing depth after hybrid capture enrichment increased strongly with the number of sequence reads obtained from a sample (r^2^ = 0.425; *p* = 0.022; Figure 2). On average, the proportion of unique virus reads decreased with enrichment steps (>99.99% shotgun, 68.92% 1×, capture, 4.94% 2× capture). The proportion of genome assembled exceeded 99% for all samples independent of the number of enrichment steps. This suggests that the virus genomes were already well covered after the first round of hybrid sequence capture and the second round provided little—if any—novel sequence information.

### 3.2. Novel Hantavirus Genome Assemblies

The large number of hantavirus sequence reads after enrichment enabled contiguous *de novo* genome assemblies from every TULV or PUUV-positive sample. The average sequence depth ranged between 247× and 12,896× per sample and genome segment with gapless contigs covering >99% of the genomes. The *de novo* assemblies were identical to partial Sanger sequences (540–750 bp) from the same rodent samples. On average, the assemblies of the S-segments had a 1.47× higher sequence read depth than the M-segments and 3.76× higher read depth than the L-segments. Sequencing depths tended to be lower at the ends of genome segments (Figure S1).

We were further able to assemble *de novo* all four TULV genomes in two common vole samples (MagDEf02, MarDSu08) naturally infected with two TULV strains each. This yielded a complete genome each of the phylogenetic clades TULV CEN.S and TULV EST.S (sequence divergence 0.16). Each assembly of each sample was fully consistent with earlier partial Sanger sequencing data [26]. There was no evidence for the existence of additional minor sequence variants or potential quasispecies in the samples.

We also examined whether fewer virus sequence reads (i.e., from lower enrichment proportions or multiplexing of more samples) could be sufficient for the genome assemblies as well by generating four random subsamples of the data from the HiSeq library with the fewest reads (13.9 × 10^6^ total reads). This yielded 2,932,625 to 3,024,231 sequence reads after quality filtering and deduplication per subsample. Of these sequence reads, 40,342 to 41,045 were virus reads (1.37% on average) that allowed each *de novo* assemblies of all genome segments with an average read depth of 497× to 506× and 99.12–99.26% genome coverage.

### 3.3. Phylogenetic, Recombination and Isolation-By-Distance Analyses

Phylogenetic analyses of the concatenated CDS showed the expected deep separation between the two orthohantavirus species and further structuring within TULV and PUUV with generally very high posterior probabilities of node support. Within TULV, the sequence from Turkey was basal to the large clades TULV CEN.S and TULV EST.S from Central Europe (Figure 1B). Multidimensional scaling of the genome sequences showed distinct clusters of PUUV and TULV samples along axis 1 (Figure S2). Consistent with the phylogeny, TULV clades and the Turkey sequence were further separated along axis 2, while PUUV sequences clustered much closer to each other. There was no consistent evidence across methods for recombination or reassortment between hantavirus species, within PUUV or between clades within TULV. However, a recombination breakpoint between the M- and L-segments was detected between two TULV CEN.S genomes (T11 and T12) next to the contact between TULV clades. This suggests a reassortment event and is consistent with earlier results on potential reassortment between M- and L-segments within TULV clades in this geographic region [26]. Isolation-by-distance analyses revealed a very strong increase in genetic distance within each hantavirus species over spatial distances of less than 500 km that levelled off at genetic distances between 0.15 and 0.2 or for branch lengths between 0.4 and 0.5 for comparisons at larger spatial distance (Mantel test: *p* = 0.001; Figure S3). Pairwise sequence comparisons between the hantavirus species were all in the genetic distance range of 0.26–0.28 or 1.1–1.4 for branch lengths, irrespective of the spatial distance between sampling locations.

### 3.4. Nucleotide Diversity and Pattern along the Genome

**Figure 3:**
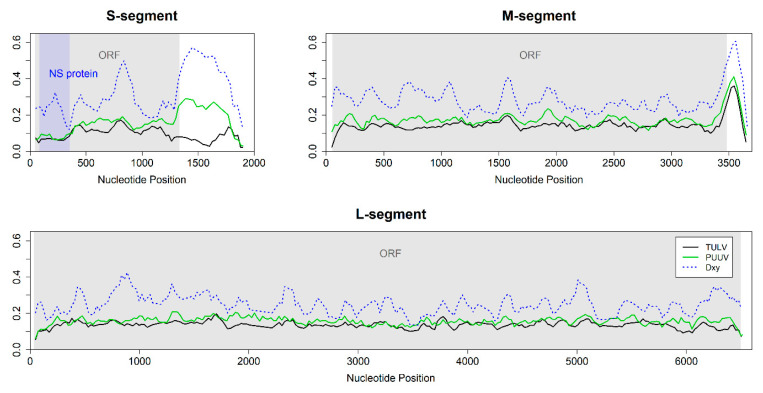
Sliding window analyses of nucleotide diversity (Pi) and the average number of nucleotide substitutions per site (D_XY_, blue line) between 20 TULV and 12 PUUV hantavirus genomes (window size 100 nt, step size 25 nt). The coding region/open reading frame (ORF) is indicated in grey. Genomic landscapes were similar between TULV and PUUV for the M- and L-segment but differed in the non-coding region of the S-segment. The nucleotide diversity within both TULV (black line) and PUUV (green line) was lower in the region encoding the non-structural (NS) protein (blue area) compared to the rest of the genome.

Hantavirus genomes of both species showed similar levels and patterns of genetic diversity in the CDS (Figure 3). The most-conserved region of the CDS was the beginning of the S-segment where a non-structural protein is encoded in a second overlapping reading frame. The large non-coding region of the S-segment showed low nucleotide diversity among TULV but high diversity among PUUV. The two species showed highest differentiation (D_XY_) in this region and in the non-coding region of the M-segment. Overall, absolute and net divergence between TULV and PUUV were highest in the S-segment and lowest for the L-segment (Table S3).

The overall nucleotide diversity differed between genomic segments (Table S3). The nucleotide diversity in S-segments was lower than in M- and L-segments. The M-segment showed the highest nucleotide diversity, irrespective of whether the entire genomes or only the CDS were considered (Table S3; Figure 3).

## 4. Discussion

Obtaining genome-wide virus sequence data from samples taken in the field or from other source materials can be associated with significant technical challenges and efforts. Here, we applied hybrid sequence capture to samples from the natural rodent hosts of TULV and PUUV and obtained complete genomes with high sequence depth that allowed extensive analyses of genomic diversity patterns. Our study complements work on other virus taxa where hybrid sequence capture was successfully used to enrich for the sequencing target (e.g., [7,10,15,16]). The method provides the advantage of higher efficiency overall compared to direct shotgun sequencing of total RNA libraries or multiplexed conventional PCR and Sanger sequencing and may be less expensive and laborious per sequenced genome [8].

There are several parameters where hybrid sequence capture protocols can be adjusted and optimized depending on the specific application and source material. A specific bait-design can contain tens of thousands of individually designed capture bait sequences. We targeted specifically closely related European hantaviruses by using mostly TULV sequences for the bait design, much fewer PUUV sequence information and only little sequence information from another hantavirus taxon, i.e., PHV (Table S2). This resulted in contiguous genome assemblies of TULV and PUUV with high sequence quality (see below). A taxonomically more diverse bait design might be useful to detect and enrich a high number of different virus species with the same design [19], an approach of which resequencing microarrays also make use of [8,18]. In contrast to microarray capture, hybrid sequence capture does not require highly specialized equipment and a very large input sequencing library. Probes are present in excess over the template and therefore enrich more efficiently for the target if the input is limited [46]. The pooling of sequencing libraries is the step where strong reductions in costs and handling time per genome can be achieved. Hybrid capture enrichment of a pool of sequencing libraries leads to straightforward reductions. However, we chose to enrich the libraries individually in independent reactions so that different virus samples could not influence each other because they might differ in virus loads or bait specificity. Further testing would be required to assess the extent of the benefits of this precaution.

In our study, separate sequence capture enrichment from single- and double-infected natural samples and the pooling of libraries for sequencing enabled the *de novo* assembly of each hantavirus genome. This is an important point because reference-based genome assemblies can be biased towards the reference sequence [47], an issue that can be avoided by *de novo* assembly methods [48,49]. Multiply infected samples pose a particular challenge to proper sequence assemblies of the respective virus strains. In our case, two voles from different locations were infected with different TULV strains, of which one each belonged to a separate phylogenetic clade within TULV (for details see [26]), a level of sequence divergence that is much lower than, e.g., between TULV and PUUV (Figure 3; Table S4). It remains to be assessed what level of sequence divergence between strains is necessary for the successful *de novo* assembly of the respective genomes. Among other factors, this will depend on the genomic distribution of sequence differences between the involved strains (Figure 3 and below) and the amount of sequence data available for assembly. However, even one fourth of the available data after hybrid sequence capture was sufficient in our study to allow contiguous and correct *de novo* assemblies as confirmed with partial Sanger sequence data. The accuracy of our hantavirus genome assemblies was further supported by phylogenetic clustering that is consistent with earlier analyses based on much shorter genome fragments obtained with Sanger sequencing (e.g., [24,26]). The very strong node support in the phylogeny suggests very few conflicting positions in the dataset. Furthermore, genetic similarity within both virus species is strongly related to spatial proximity resulting in the expected isolation-by-distance pattern within and no association of genetic divergence and geographic distance between the species (Figure S3, [24]). Despite pervasive mutational saturation that limits the estimated level of divergence between more distant strains within species (Figure S3; see [24]), access to full genome data holds thus the potential for a better resolution of the phylogenetic relationships and evolutionary history within and between European hantaviruses and others (Figure 1).

Our array of complete genomes revealed also similar nucleotide diversity patterns (genomic landscapes) along TULV and PUUV sequences (Figure 3). Similarities in genomic landscapes between species were demonstrated in vertebrates as well (e.g., [50]). It has been suggested that they could be a result of linked selection and recombination rate variation along the vertebrate genomes that causes reduced geneflow [51], or of other yet unknown genomic mechanisms [52]. The frequency of recombination in TULV and PUUV hantaviruses is probably low as our and other analyses suggest [26] but the analysis of more full-genome datasets will be necessary for a comprehensive picture. However, recombination in hantaviruses is certainly much lower than in vertebrate genomes [53,54] making variation in its rate an unlikely explanation for correlated genomic landscapes. We consider it thus more likely that genomic landscapes in hantaviruses are a consequence of intrinsic selective constraints in sequence evolution related to the encoded function of the genome segments. This would lead to correlated patterns of nucleotide variation within species but not necessarily to associated patterns in divergence between species.

Nucleotide diversity of both TULV and PUUV was highest in the M-segment but the S-segment showed the largest divergence between species associated with the lowest diversity within species (Table S3). High sequence diversity in the M-segment is consistent with adaptive considerations because the encoded surface proteins interact directly with the host cell [21] and are thus likely to show an adaptive response to variation in the hosts (e.g., [26,55]). The L-segment, encoding the RNA-dependent RNA polymerase, is not only the least divergent segment between TULV and PUUV but also relatively conserved between other hantavirus species [56] although nucleotide diversity within the CDS reaches almost the levels of the M-segment.

In comparison, nucleotide diversity within the CDS of the S-segment is much lower than in the other segments and sequence divergence is particularly high (Figure 3; Table S3). This could indicate potential functional differences between the S-segments of TULV and PUUV. The peak of divergence at approximately nucleotide position 800 (Figure 3) suggests this section of the S-segment as the most promising for future research into functional differences between hantavirus species. The hantavirus nucleocapsid protein encoded in the S-segment interacts with the other viral proteins to regulate virus assembly and replication [57,58], regulates the function of ribosomal proteins to enhance the translation of viral mRNA [59,60] and interferes with the host immune system by downregulating apoptosis [61] and inhibiting interferon signaling response [62]. The non-structural protein encoded in a second, overlapping reading frame in the S-segment was suggested to function as a weak interferon inhibitor [63]. Mutations in overlapping reading frames often damage several distinct functions simultaneously [64]. Therefore, purifying the selection in the overlapping reading frame of the S-segment is expected to be particularly strong and consistent with the observed reduction in nucleotide diversity in this part of the CDS (Figure 3).

Sequence divergence between TULV and PUUV is also particularly high in the non-coding region of the S-segment. This pattern is associated with remarkable differences in nucleotide diversity between species (Figure 3). The conservation of this non-coding region in TULV could indicate, e.g., functionally important secondary structures in this species, but not in PUUV (see [65]). Non-coding regions are often not included in published (partial) genome sequences of orthohantaviruses and other taxa, which may be, at least in part, because of technical challenges. However, the increased efficiency of high-throughput sequencing methods is likely to change this in the near future and may thus provide a more complete understanding of the genome-wide sequence variation in hantaviruses in ecological and evolutionary settings.

## Figures and Tables

**Table 1:** List of TULV and PUUV high-throughput sequencing-based genome assemblies analyzed in this study. The number of capture rounds (zero: shotgun; one or two enrichment steps), the sequencing instrument used, number of total sequence reads, unique sequence reads (number of reads after filtering duplicates), mapped virus reads (deduplicated reads post-mapping) and the average genome sequence depth are shown. Shotgun sequence read data are from [26].

	Capture	Sequencer	Total Reads	Unique Reads	Virus Reads	Mean Sequence Depth
**Tula virus**						
T10 MarCzKa04	0	HiSeq	30,240,364	30,239,770	1737	9
T9 MarDGb22	0	HiSeq	43,470,283	43,469,619	1781	8
T18 MarDKbB31	0	HiSeq	92,841,279	92,824,579	26,721	48
T19 MarDPf01	0	HiSeq	65,793,875	65,787,860	12,006	34
T8 MarDRb01	0	HiSeq	57,580,789	57,571,558	16,077	38
T13 MarDHg01	0	MiSeq	11,563,256	11,563,254	1539	30
T3 MarDDh05	0	MiSeq	12,157,472	12,157,461	1712	34
T4 MarCzHo09	0	MiSeq	10,363,464	10,363,453	1850	38
T5 MarCzJe04	0	MiSeq	12,634,308	12,634,294	1974	38
T15 MarDSp01	0	MiSeq	2,286,266	2,286,252	4766	97
T14 MarDOt03	0	MiSeq	11,907,702	2,286,176	4682	91
T17 MarDSq15_1 ^1^	0	HiSeq	53,177,359	53,177,238	583	4
T17 MarDSq15_2 ^1^	1	HiSeq	25,564,658	22,901,877	515,194	6238
T6 MarCzGr07	1	HiSeq	13,928,878	10,770,028	62,939	771
T2/T12 MarDSu08_1 ^1^	1	HiSeq	16,711,898	12,660,735	184,258	2249
T2/T12 MarDSu08_2 ^1^	2	HiSeq	28,180,378	1,069,556	334,893	4068
T2/T12 MarDSu08_3 ^1^	1	MiSeq	1,812,673	1,812,673	19,035	378
T1/T11 MagDEf02_1 ^1^	2	HiSeq	30,333,274	1,168,089	734,578	8780
T1/T11 MagDEf02_2 ^1^	2	HiSeq	31,420,140	1,099,805	436,943	5267
T16 MarCHEl42	2	HiSeq	19,306,058	1,666,013	1,066,770	12,896
**Puumala virus**						
P9 MglDCr02	1	MiSeq	2,914,826	1,779,417	57,070	1692
P11 MglDKe04	1	MiSeq	2,243,640	1,160,159	19,312	413
P12 MglDKe05	1	MiSeq	2,702,868	1,374,479	12,927	247
P7 MglLTU01	1	MiSeq	2,676,336	1,719,083	152,509	3004

^1^ Technical replicates.

## Supplementary Materials

The following are available online at https://www.mdpi.com/1999-4915/12/7/749/s1. Table S1: List of all orthohantavirus genome sequences analyzed in this study, Figure S1: Average relative sequence read depth of 17 TULV and four PUUV genome assemblies, Figure S2: Multidimensional scaling of pairwise genetic distance between virus genomes, Figure S3: (A) Pairwise genetic distances between genome sequences and (B) branch lengths extracted from Bayesian phylogenetic reconstruction plotted against pairwise geographic distances, Table S2: Sequences used as references for hybrid sequence capture bait design. GeBank accession numbers for each sequence are given, Table S3: Estimates of average evolutionary divergence over sequence pairs within and between orthohantavirus species, Table S4. Genetic distances between technical replicates of samples or genomes from the same or different sampling localities, Table S5.
