# Supplementary material for: Assessing Genome-Wide Diversity in European Hantaviruses through Sequence Capture from Natural Host Samples
Source: Viruses. 2020 Jul 11;12(7):749. doi: 10.3390/v12070749 (PMC7412162; doi:10.3390/v12070749)
Supplement: Supplementary file 1 [file viruses-12-00749-s001.zip › 5-viruses-832211-suppl proof done/Supplementary_viruses_2_GH.pdf]

viruses
MDPI

Table S1. List of all orthohantavirus genome sequences analyzed in this study.

	Location	Country	Latitude	Longitude	Reference	GenBank accession number		
**Tula virus**						**S-Segment**	**M-Segment**	**L-Segment**
T1 MagDEf02_1[2]	Elisenfels	Germany	50.040	12.168	this study	MT514275	MT514285	MT514295
T2 MarDSu08_1[2]	Seussen	Germany	50.031	12.161	this study	MT514279	MT514289	MT514299
T3 MarDDh05	Dornhof	Germany	50.026	12.203	[1]	MK386130	MK386142	MK386154
T4 MarCzHo09	Horni Lomany	Czech Republic	50.135	12.340	[1]	MK386131	MK386143	MK386155
T5 MarCzJe04	Jeronym	Czech Republic	50.100	12.736	[1]	MK386132	MK386144	MK386156
T6 MarCzGr07	Grygov	Czech Republic	49.540	17.330	this study	MT514278	MT514288	MT514298
T7 Moravia	Moravia	Czech Republic	49.159	16.679	[2–4]	NC_005227	NC_005228	NC_005226
T8 MarDRb01	Regenbogen	Germany	49.212	12.710	[1]	MK386140	MK386152	MK386164
T9 MarDGb22	German Border	Germany	49.331	12.969	[1]	MK386137	MK386149	MK386161
T10 MarCzKa04	Kanicky	Czech Republic	49.460	13.177	[1]	MK386136	MK386148	MK386160
T11 MagDEf02_2[2]	Elisenfels	Germany	50.040	12.168	this study	MT514276	MT514286	MT514296
T12 MarDSu08_2[2]	Seussen	Germany	50.031	12.161	this study	MT514280	MT514290	MT514300
T13 MarDHg01	Hildbrandsgrün	Germany	50.209	11.733	[1]	MK386133	MK386145	MK386157
T14 MarDOt03	Oberthölau	Germany	50.034	12.094	[1]	MK386134	MK386146	MK386158
T15 MarDSp01	Spielberg	Germany	50.168	12.039	[1]	MK386135	MK386147	MK386159
T16 MarCHEl42	Ersigen	Switzerland	47.110	7.597	this study	MT514277	MT514287	MT514297
T17 MarDSq15[1]	St. Quirin	Germany	49.118	12.531	this study, [1]	MK386141	MK386153	MK386165
T18 MarDKbB31	Knöbling B	Germany	49.170	12.619	[1]	MK386138	MK386150	MK386162
T19 MarDPf01	Pfaffenthann	Germany	49.078	12.388	[1]	MK386139	MK386151	MK386163
T20 Turkey	Palandoken	Turkey	39.826	41.279	[5]	MH649270	MH649271	MH649272
**Puumala virus**								
P1 Kazan	Kazan	Russia	55.800	49.105	[6,7]	Z84204	Z84205	EF405801
P2 Samara49	Samara49	Russia	53.436	49.665	[8]	AB433843	AB433850	AB574183
P3 Samara94	Samara94	Russia	53.363	50.254	[8]	AB433845	AB433852	AB574184
P4 Ufa	Ufa	Russia	54.739	56.485	[9]	AB297665	AB297666	AB297667
P5 Pieksamaki	Pieksamaki	Finland	62.300	27.133	[10]	JN831943	JN831944	JN831945
P6 Sotkamo	Sotkamo	Finland	63.900	28.416	[11,12]	NC_005224	NC_005223	NC_005225
P7 MglLTU01	Lukstas	Lithuania	55.850	26.200	this study, [13]	MT514274	MT514284	MT514294

Table S1. *Cont.*

	Location	Country	Latitude	Longitude	Reference	GenBank accession number		
**Puumala virus**						**S-Segment**	**M-Segment**	**L-Segment**
P8 Umea	Umea	Sweden	63.749	20.230	[14]	AY526219	AY526218	AY526217
P9 MglDCr02	Crailsheim	Germany	49.133	10.063	this study, [15]	MT514271	MT514281	MT514291
P10 Astrup	Astrup	Germany	52.284	8.278	[16]	KJ994776	KJ994777	KJ994778
P11 MglDKe04	Kenzingen	Germany	48.194	7.769	this study, [15]	MT514272	MT514282	MT514292
P12 MglDKe05	Kenzingen	Germany	48.194	7.769	this study, [15]	MT514273	MT514283	MT514293
P13 Ardennes_Mg75	Ardennes	France	49.761	4.628	[17]	KT247593	KT247602	KT247608
P14 Ardennes_Mg156	Ardennes	France	49.761	4.628	[17]	KT247592	KT247603	KT247609
P15 Orleans_Mg23	Orleans	France	47.902	1.909	[17]	KT247594	KT247600	KT247604
P16 Orleans_Mg29	Orleans	France	47.902	1.909	[17]	KT247595	KT247601	KT247605
P17 Jura_Mg2	Jura	France	46.916	6.229	[17]	KT247596	KT247598	KT247606
P18 Jura_Mg214	Jura	France	46.916	6.229	[17]	KT247597	KT247599	KT247607

[1] Technical replicate, see main text. [2] Multiple infection (see main text).

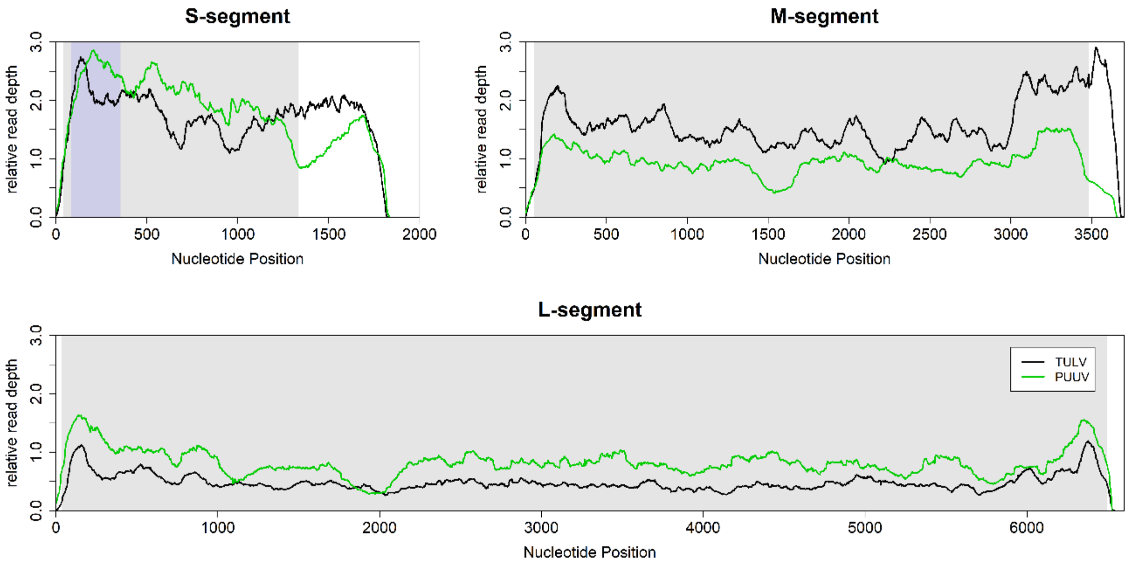

Figure S1. Average relative sequence read depth of 17 TULV (black) and four PUUV (green) genome assemblies. Details for effective sequence read depth per genome are given in Table 1. The coding region / open reading frame (ORF) is indicated in grey.

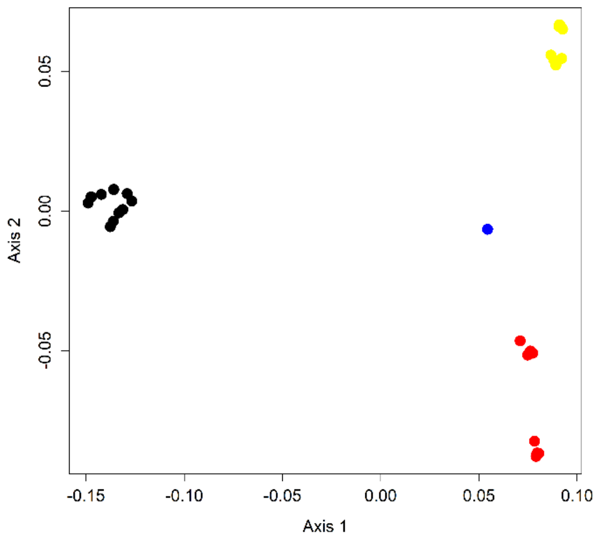

Figure S2. Multidimensional scaling of pairwise genetic distance between virus genomes. Virus species cluster separately – PUUV (black, left) and TULV (right) – but there is also distinct clustering between TULV clades (CEN.S: red, EST.S: yellow, Turkey: blue).

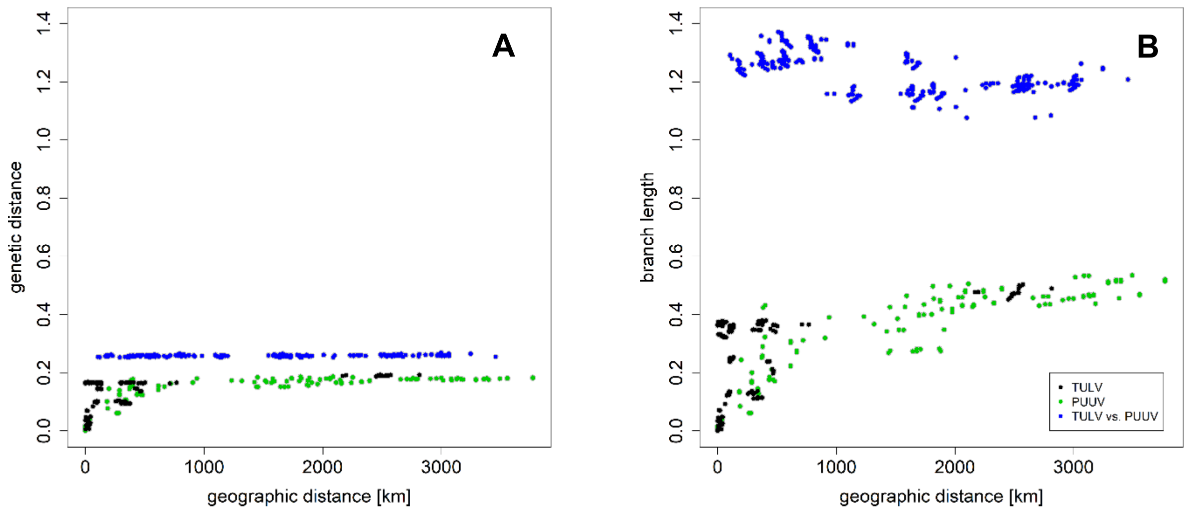

Figure S3. (**A**) Pairwise genetic distances between genome sequences and (**B**) branch lengths extracted from Bayesian phylogenetic reconstruction plotted against pairwise geographic distances. Genetic divergence shows strong isolation-by-distance relationships within each hantavirus species until mutational saturation limits sequence divergence for geographic distances larger than approximately 500 km. Substitution models in phylogenetic reconstruction correct for mutational saturation only partially (see [18] for details). Between species comparisons show a clearly higher level of sequence divergence irrespective of geographic distance.

Table S2. Sequences of TULV, PUUV and Adler virus used as references for hybrid sequence capture bait design. GenBank accession numbers for each sequence are given.

	S	M	L
**Tula virus**			
Moravia	NC005227.2	NC005228	NC005226.1
Dornhof	MK386130	MK386142	MK386154
Horni Lomany	MK386131	MK386143	MK386155
German border	MK386137	MK386149	MK386161
Regenbogen	MK386140	MK386152	MK386164
Kanicky	MK386136	MK386148	MK386160
Perwolfing	MK376675.1		
Spielberg	MK386135	MK386147	MK386159
Oberthoelau	MK386134	MK386146	MK386158
Pfaffenthann	MK386139	MK386151	MK386163
St. Quirin	MK386141	MK386153	MK386165
Knöbling B	MK386138	MK386150	MK386162
Elisenfels	MK376603.1	MK376565.1	MK376478.1
Aveyron	SEG123		
Lodz	AF063892	AF063891.2	
Velika Goria	AF164094		
Sennickerode	EU439947		
Scharfenberg		HQ728439	HQ728453.1
Marzehns	DQ662094	DQ768149.1	
Biebersdorf			HQ728460.1
Eberswalde	DQ768143	DQ768144.1	
Brandenburg	DQ665812.1		
Ravanica	KF557547	KF177178.1	
Cacak	AF017659	AF017658.1	
Kosice	Y13979		
Kosice	Y13981		
Tula	Z30941	Z48576.1	
Karatal	AM945877		
Krim	KJ742927.1	KJ742933.1	KJ742931.1
**Puumala virus**			
Sokamo	NC_005224.1	NC_005223.1	NC_005225.1
Astrup	KJ994776	KJ994777.1	KJ994778.1
Jura	KT247596.2	KT247598.1	KT247606.1
Samara	AB433843.2	AB433850.2	AB574183.1
**Adler virus**	KP013572.1	KP013581.1	KP013560.1
**Prospect Hill virus**	M34011.1	X55129.1	EF646763.1

Table S3. Estimates of average evolutionary divergence over sequence pairs within and between orthohantavirus species calculated in MEGA7.

Divergence	TULV	PUUV	Between species	Net between species
**Genome**				
S	0.094	0.154	0.306	0.182
M	0.143	0.173	0.281	0.123
L	0.137	0.156	0.246	0.100
Full genome	0.132	0.161	0.266	0.119
**CDS**				
S	0.105	0.139	0.265	0.143
M	0.140	0.162	0.274	0.123
L	0.138	0.152	0.248	0.103
Full CDS	0.135	0.154	0.258	0.114

Table S4. Genetic distances between technical replicates of samples or genomes from the same or different sampling localities (see Table S1).

	Virus	p-distance	Comparison between
**Technical replicates**			
MarDSq15	TULV	0.00067	Shotgun – 1x capture
MarDSu08_ES	TULV	0	Library 1 – Library 2
MarDSu08_CS	TULV	0	Library 1 – Library 2
MarDSu08	TULV	0 (0.00075[1])	MiSeq – HiSeq
MagDEf02_ES	TULV	0	Library 1 – Library 2
MagDEf02_CS	TULV	0	Library 1 – Library 2
**Locations**			
DSu-DEf_ES	TULV	0.00522	1.2 km distance
DSu-DEf_CS	TULV	0.03840	1.2 km distance
MglDKe	PUUV	0.00008	Same location
Jura	PUUV	0.01756	Same location
Ardennes	PUUV	0.01613	Same location
Orleans	PUUV	0.01212	Same location

[1] Reference-based mapping.

References

1. Saxenhofer, M.; Schmidt, S.; Ulrich, R.G.; Heckel, G. Secondary contact between diverged host lineages entails ecological speciation in a European hantavirus. *PLoS Biol.* **2019**, *17*, e3000142.
2. Plyusnin, A.; Vapalahti, O.; Lankinen, H.; Lehväslaiho, H.; Apekina, N.; Myasnikov, Y.; Kallio-Kokko, H.; Henttonen, H.; Lundkvist, A. Tula virus: A newly detected hantavirus carried by European common voles. J. Virol. 1994, 68, 7833–7839.
3. Vapalahti, O.; Lundkvist, Å.; Kukkonen, S.K.; Cheng, Y.; Gilljam, M.; Kanerva, M.; Manni, T.; Pejcoch, M.; Niemimaa, J.; Kaikusalo, A.; et al. Isolation and characterization of Tula virus, a distinct serotype in the genus Hantavirus, family Bunyaviridae. J. Gen. Virol. 1996, 77, 3063–3067.
4. Kukkonen, S.K.; Vaheri, A.; Plyusnin, A. Completion of the Tula hantavirus genome sequence: Properties of the L segment and heterogeneity found in the 3'termini of S and L genome RNAs. J. Gen. Virol. 1998, 79, 2615–2622.
5. Polat, C.; Ergünay, K.; Irmak, S.; Erdin, M.; Brinkmann, A.; Çetintaş; O.; Çoğal, M.; Sözen, M.; Matur, F.; Nitsche, A.; et al. A novel genetic lineage of Tula orthohantavirus in Altai voles (*Microtus obscurus*) from Turkey. *Infect. Genet. Evol.* **2019**, *67*, 150–158.
6. Nemirov, K.; Vapalahti, O.; Papa, A.; Plyusnina, A.; Lundkvist, Å.; Antoniadis, A.; Vaheri, A.; Plyusnin, A. Genetic characterization of new Dobrava hantavirus isolate from Greece. *J. Med. Virol.* **2003**, *69*, 408–416.

7. Lundkvist, A.; Cheng, Y.; Sjölander, K.B.; Niklasson, B.; Vaheri, A.; Plyusnin, A. Cell culture adaptation of Puumala hantavirus changes the infectivity for its natural reservoir, Clethrionomys glareolus, and leads to accumulation of mutants with altered genomic RNA S segment. J. Virol. 1997, 71, 9515–9523.
8. Seto, T.; Tkachenko, E.A.; Morozov, V.G.; Tanikawa, Y.; Kolominov, S.I.; Belov, S.N.; Nakamura, I.; Hashimoto, N.; Kon, Y.; Balakiev, A.E.; et al. An efficient in vivo method for the isolation of Puumala virus in Syrian hamsters and the characterization of the isolates from Russia. J. Virol. Methods 2011, 173, 17–23.
9. Abu Daud, N.H.; Kariwa, H.; Tkachenko, E.; Dzagurnova, T.; Medvedkina, O.; Tkachenko, P.; Ishizuka, M.; Seto, T.; Miyashita, D.; Sanada, T.; et al. Genetic and antigenic analyses of a Puumala virus isolate as a potential vaccine strain. Jpn. J. Vet. Res. 2008, 56, 151–165.
10. Plyusnina, A.; Razzauti, M.; Sironen, T.; Niemimaa, J.; Vapalahti, O.; Vaheri, A.; Henttonen, H.; Plyusnin, A. Analysis of complete Puumala virus genome, Finland. Emerg. Infect. Dis. 2012, 18, 2070.
11. Vapalahti, O.; Kallio-Kokko, H.; Salonen, E.M.; Brummer-Korvenkontio, M.; Vaheri, A. Cloning and sequencing of Puumala virus Sotkamo strain S and M RNA segments: Evidence for strain variation in hantaviruses and expression of the nucleocapsid protein. J. Gen. Virol. 1992, 73, 829–838.
12. Piiparinen, H.; Vapalahti, O.; Plyusnin, A.; Vaheri, A.; Lankinen, H. Sequence analysis of the Puumala hantavirus Sotkamo strain L segment. Virus Res. 1997, 51, 1–7.
13. Straková, P.; Jagdmann, S.; Balčiauskas, L.; Balčiauskienė, L.; Drewes, S.; Ulrich, R.G. Puumala virus in bank voles, Lithuania. Emerg. Infect. Dis. 2017, 23, 158.
14. Johansson, P.; Olsson, M.; Lindgren, L.; Ahlm, C.; Elgh, F.; Holmström, A.; Bucht, G. Complete gene sequence of a human Puumala hantavirus isolate, Puumala Umeå/hu: Sequence comparison and characterisation of encoded gene products. Virus Res. 2004, 105, 147–155.
15. Drewes, S.; Turni, H.; Rosenfeld, U.M.; Obiegala, A.; Straková; P.; Imholt, C.; Glatthaar, E.; Dressel, K.; Pfeffer, M.; Jacob, J.; et al. Reservoir-driven heterogeneous distribution of recorded human Puumala virus cases in South-West Germany. Zoonoses Public Health 2017, 64, 381–390.
16. Ali, H.S.; Drewes, S.; Weber de Melo, V.; Schlegel, M.; Freise, J.; Groschup, M.H.; Heckel, G.; Ulrich, R.G. Complete genome of a Puumala virus strain from Central Europe. Virus Genes 2015, 50, 292–298.
17. Castel, G.; Couteaudier, M.; Sauvage, F.; Pons, J.B.; Murri, S.; Plyusnina, A.; Pontier, D.; Cosson, J.F.; Plyusnin, A.; Marianneau, P.; et al. Complete genome and phylogeny of Puumala hantavirus isolates circulating in France. Viruses 2015, 7, 5476–5488.
18. Saxenhofer, M.; Weber de Melo, V.; Ulrich, R.G.; Heckel, G. Revised time scales of RNA virus evolution based on spatial information. Proc. R. Soc. B 2017, 284, 20170857.

© 2020 by the authors. Licensee MDPI, Basel, Switzerland. This article is an open access article distributed under the terms and conditions of the Creative Commons Attribution (CC BY) license (http://creativecommons.org/licenses/by/4.0/).
